# Posterior Reversible Encephalopathy Syndrome in an Adult Male With Uncontrolled Blood Pressure and Cocaine Use: A Case Report

**DOI:** 10.7759/cureus.36824

**Published:** 2023-03-28

**Authors:** Betsy Benjamin, Bereket Tewoldemedhin, Aaron Rodas

**Affiliations:** 1 Family Medicine, Lower Bucks Hospital, Bristol, USA; 2 Internal Medicine, Lower Bucks Hospital, Bristol, USA

**Keywords:** vasogenic edema, severe hypertension, hypertension, hypertension induced pres, cocaine induced pres, posterior reversible encephalopathy syndrome (pres)

## Abstract

Posterior reversible encephalopathy syndrome (PRES) is a subacute syndrome that is diagnosed by neurologic symptoms and radiologic findings. PRES is predominantly caused by uncontrolled hypertension though it has been associated with illicit drug use, specifically cocaine use. We describe a case of a 68-year-old male who developed visual disturbances and gait abnormalities. Imaging was confirmed with head CT that showed hypoattenuation in the posterior aspects of the occipital lobes. The patient was managed with anti-hypertensive medication and blood pressure monitoring during his hospital course. Therefore, the patient's neurological symptoms resolved once the blood pressure was well-controlled. MRI of the brain was completed prior to discharge and confirmed resolution. Hypertension and cocaine use has been documented as causative agents of PRES. It is most likely due to the inability of the posterior circulation of the brain to auto-regulate with acute changes in blood pressure resulting in hypoperfusion and disruption of the blood-brain barrier with resultant vasogenic edema without infarction.

## Introduction

Posterior reversible encephalopathy syndrome (PRES) has also been termed reversible posterior leukoencephalopathy syndrome (R PLS), which is one of the neuro-autoregulatory disturbance diseases associated with acute changes in blood pressure resulting in hypoperfusion and disruption of the blood-brain barrier with resultant vasogenic edema with clinical disease.

This is a report about a patient with new onset of visual disturbances and gait abnormality with elevated blood pressure who was not managed on anti-hypertensive medications. A CT scan showed the characteristic abnormalities of PRES. Blood pressure control with anti-hypertensive resulted in a complete resolution of clinical findings. Here, we will discuss a 68-year-old male who presented to the hospital with visual disturbances.

## Case presentation

A 68-year-old African American male with a past medical history of prostate cancer (status post radiotherapy in remission five to six years ago), chronic lower back pain, retinal detachment, and whiplash injury secondary to a motor vehicle accident (3 years ago) came to the emergency department with three days of worsening visual disturbances. The patient stated he noticed intermittent blurry and double vision two months ago with associated “floaters” and “black spots.” However, three days prior to admission the visual disturbances were constant. Associated symptoms include dizziness and feeling unsteady on his feet with no falls. He stated there were no triggering events, including recent trauma or infections. He had not seen a hematologist/oncologist or primary care after completing radiation. He was not on medications prior to admission. He denied being diagnosed with hypertension or hyperlipidemia. He denied associating symptoms such as headaches, seizures, slurred speech, loss of consciousness, nausea, vomiting, diarrhea, or constipation. He admits to cigarette use of 1/2 pack per day for 40 years. He stated he had used cocaine the day of the admission but does not recall the amount he took. On review of systems, the patient was more fatigued than usual and stated he was falling asleep earlier with increased sleep latency and urinary urgency.

At the time of admission, his blood pressure was 144/116 and all other vitals were unremarkable.

On the physical exam, he was asleep but arousable to voice and was thin and dry in appearance. He was oriented to people, place, and time. The patient had acceptable speech but was slow in mentation but was cooperative. On neurological exam, cranial nerves II-XII with mild asymmetry of pupils where the right was larger than the left in the range of 1-1.5 mm. Dipoplia was not obvious. The neck was supple but painful on movement. On motor exam, motor strength was intact and toned but decreased muscle bulk throughout both lower and upper extremities. Tendon reflexes were brisk ranging from 0-1 and 1-2/4 in different muscle groups. The patient was able to finger to nose however there was a mild intention tremor on the left upper extremity. Gait was unsteady, otherwise, without gross abnormalities. Sensorium was significantly decreased to absent distally in the lower extremity bilaterally. Cardiovascular, pulmonology, and abdominal exam were unremarkable.

On labs, the patient tested positive for cocaine and opiate on the urine drug screen and all other labs were unremarkable. EKG showed normal sinus rhythm, 76 beats per min with left ventricular hypertrophy with no ST and T-wave abnormalities. All other cardiac workup was unremarkable.

**Figure 1:**
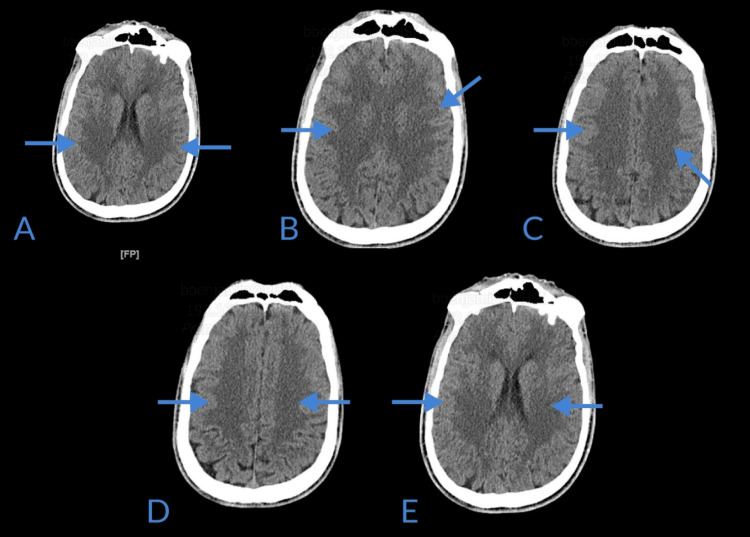
Computer tomography of the head without contrast demonstrating focal symmetric areas of hypo-attenuation (blue arrows) in the parieto-occipital regions (A, B, C, D, E)

Head computer tomography (CT) showed focal symmetric areas of prominent hypoattenuation in the posterior aspects of the occipital lobes bilaterally, as well as parietal regions, indicative of posterior reversible encephalopathy syndrome (Figures 1A-1E).

Mild, chronic microangiopathic ischemic changes in the white matter with intracranial atherosclerosis were also seen. The patient was started on amlodipine 10 mg on admission, neurology was consulted, and the patient was monitored on the telemetry floor. The following day, the patient noted that the visual disturbances continued with double vision and floaters. His blood pressure continued to be elevated, and he was started on hydrochlorothiazide.

Neurology recommended magnetic resonance imaging (MRI) of the brain for confirmation and an electroencephalogram (EEG) to evaluate for seizures. EEG was completed, which noted delta waves compatible with underlying cerebral dysfunction as well as rhythmic theta activity arising from the bilateral head region compatible with a seizure disorder. MRI was attempted on the second day of admission, but it was a severely limited study secondary to pain and the patient was unable to continue with the study.

**Figure 2:**
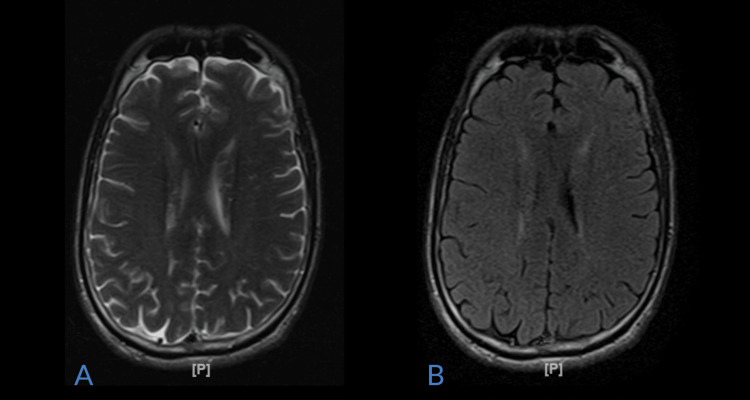
T2 axial (A) and axial flare (B) magnetic resonance imaging demonstrating no vasogenic edema in the parieto-occipital regions on the third day of hospitalization

On the third day of admission, there was some improvement in visual disturbances, however, the patient had a loss of appetite. His blood pressure then stabilized to 120/84 on the third day of admission with a resolution of symptoms. MRI of the head was completed on the third day, which showed no signs of intracranial abnormalities, masses, or enhancements (Figures 2A-2B).

In the majority of patients with posterior reversible encephalopathy syndrome described in the literature, it is associated with systemic diseases such as seizure disorders, end-stage renal disease, autoimmune disorders, pre-eclampsia, or cocaine use. This case presents an atypical patient who had a gradual onset of visual disturbances with cocaine use and EEG confirming possible seizure. Symptoms resolved after initiating anti-hypertensive medication such as amlodipine. Further evaluation in the outpatient setting was recommended by neurology for the first-time seizure. MRI of the brain was completed after blood pressure stabilized and showed no signs of PRES. 

Even though the exact mechanism of PRES remains unknown, it can be observed that it is due to an altered blood-brain barrier because of elevated blood pressure with endothelial injury. The clinical presentation is nonspecific, with no well-defined diagnostic criteria; therefore; clinical suspicion is based on imaging studies to make for proper management.

## Discussion

Posterior reversible encephalopathy syndrome (PRES) has also been called reversible posterior leukoencephalopathy syndrome (RPLS), which is a neurotoxic disease associated with acute onset of symptoms, usually neurologic, with characteristic imaging changes of patchy vasogenic edema within the brain parenchyma [1,2]. It is thought to arise from the inability of the posterior circulation of the brain to autoregulate to acute changes in blood pressure, resulting in hypoperfusion and disruption of the blood-brain barrier with resultant vasogenic edema without infarction [1-3]. These findings tend to be associated with elevated blood pressure, fluid overload, or sudden fluctuations in renal function [3,4]. Most commonly, the syndrome has been seen to affect the parieto-occipital regions of the brain, making it distinctly separate from chronic hypertensive encephalopathy with microangiopathy, which tends to affect the basal ganglia, pons, and cerebellum [3,4]. A study that examined 31 patients who were diagnosed with PRES showed that 87% of patients presented with a dominant parieto-occipital pattern [3]. Clinical presentation can be extremely varied from headaches, seizures, encephalopathy, and visual disturbances with reversible cortical blindness [2-4]. Also, patients can present with ataxia, vertigo, and tinnitus [2,3]. 

The syndrome usually affects adults and rarely children where most cases have been reported to occur between the ages of 20 and 65 [1,2]. Overall, the incidence is unknown but it is more common in females and is associated with pregnancy and autoimmune diseases [2,3]. Multiple risk factors have been postulated including acute as well as chronic kidney disease, systemic lupus erythematosus, polyarteritis nodosa, HIV infection, sepsis, hemolytic uremic syndrome, and thrombotic thrombocytopenic purpura [2]. Rarely, the syndrome has been reported due to pre-eclampsia [3,4]. Risk factors that predispose to sudden fluctuations of blood pressure such as endocrine disorders with pheochromocytoma, primary aldosteronism, porphyria, and substance use disorder (especially cocaine and amphetamines), severe electrolyte derangements, including hypercalcemia and hypomagnesemia, have been also postulated as risk factors for PRES [2,3]. Immunosuppressive and immunomodulatory therapies for malignancy, transplant, and rheumatologic conditions, including cisplatin, cyclosporin, gemcitabine, bevacizumab, vascular endothelial growth factor (VEGF) inhibitors, methotrexate, and tacrolimus to a lesser extent, can lead to the syndrome [4-6].

The exact pathophysiology remains elusive and proposed theories include the disruption of vascular auto-regulation as described above [1-3]. Other mechanisms implicated include endothelial dysfunction especially as seen with conditions like preeclampsia and cytotoxic therapies that cause increased capillary leakage with disruption of the blood-brain barrier and axonal swelling with resultant vasogenic edema [2,3,7]. Markers of endothelial dysfunction have been noted in these patients, including elevated endothelin-1, tissue plasminogen activator, fibronectin levels, and specifically von Willebrand's factor. These markers support the fact that endothelial injury is part of the pathophysiology [5-7].

The clinical presentation of patients with PRES tends to have varied scenarios but characteristic findings include an acute onset of headache in 50%, confusion with altered mental status in 50%-80%, visual disturbance with diplopia as well as loss of vision in more than 30%, and seizures, either focal or generalized, in more than 60% [2-4]. In women, pregnancy always needs to be ruled out in the appropriate clinical setting. Notable physical findings include visual neglect in more than 40%, cortical blindness and brisk reflexes with a lack of coordination in more than 30% while a small minority can present as stuporous or comatose [2,3,7].

The diagnosis of PRES syndrome largely relies on a combination of clinical findings in the appropriate clinical setting aided by neuro-diagnostic imaging [4,7]. A non-contrast head computed tomography (CT) scan is usually the initial evaluation performed especially in the emergency department [1,2]. Typical findings would include bilateral white matter edema within the posterior cerebral hemispheres, particularly in the parieto-occipital regions [1,8]. Characteristic findings include sparing of the calcarine and paramedian parts of the occipital lobe, which helps distinguish PRES from bilateral posterior cerebral infarctions [6-8]. Other involved areas include the cerebellum (40%) and brainstem (18%). Predominant posterior fossa lesions while uncommon can occur and certain variants of PRES hence making CT scans unreliable in identifying these lesions [6-8].

Magnetic resonance imaging (MRI) is the diagnostic modality of choice [7-9]. Characteristic lesions include hyper-intense signals on T2-weighted images on affected areas [8,9]. Septal peripheral lesions and cortical lesions are better detected on fluid-attenuated inversion recovery (FLAIR) sequences [7,8]. With the administration of gadolinium: disruption of the blood-brain barrier can be better visualized, appearing as gyriform signal enhancement and nodular subcortical enhancement [7,9]. Diffusion-weighted imaging (DWI) aids to differentiate PR ES from basilar strokes [6-8]. In PR ES the vasogenic edema would appear as a hypo or iso-intense signal on DWI with occasional slightly hyper-intense images due to the T2 shine-through effect [7,8]. An additional increased signal on apparent diffusion coefficient (ADC) maps is also seen [7-9]. This is in stark contrast to acute cerebral infarcts, which would produce marked hyperintensity on DWI images and hypo-intensity on ADC maps [7-9].

Other tests that are performed, but non-diagnostic, could aid in elucidating underlying risks, including metabolic as well as toxic screening for renal dysfunction and the identification of chemical imbalance [1,2]. EEG can be used to rule out status epilepticus in patients who come in with seizures [4,5]. Lumbar puncture, although not required in the evaluation of most patients, if performed to rule out metastatic malignancies and meningoencephalitis would typically show cerebrospinal fluid with slightly elevated levels of albumin and elevated CSF to serum albumin ratio with no pleocytosis and no white blood cell count indicating disruption of the blood-brain barrier without an inflammatory process [7-9].

The management of PR ES syndrome is multipronged and involves focused treatment on lowering blood pressure, removal of the precipitating cause, temporary stabilization of underlying disorders, including aggressive correction of electrolyte derangements, and management of seizures with prophylaxis [9,10]. Hypertension is the predominant feature and the majority of patients with PRES regardless of the etiology improve symptoms dramatically with a lowering of blood pressure [9,10]. Goal blood pressures are not defined and are dependent on the patient's baseline to a large extent [10,11]. The blood pressure-lowering regimen follows the American College of Cardiology guidelines with the aim of reducing diastolic blood pressure to approximately 100 to 105 mmHg to be achieved within two to six hours with the maximum fall not to exceed 25% of the presenting value of the patient [9,10]. In patients presenting with hypertensive emergencies, oral therapy continues to be discouraged and IV titratable antihypertensives, including nicardipine, clevidipine, and labetalol are preferred [12]. Medication selection should be patient-specific and particular contraindications need to be sought out while selecting antihypertensives, depending on underlying factors [12]. Concomitant underlying disorders, including sepsis, preeclampsia, and autoimmune disorder exacerbations, need to be identified and managed [9-11].

Seizure prophylaxis and treatment should be instituted in non-pregnant patients presenting with seizures by the use of a standard seizure protocol with IV anti-epileptics [8,9]. Selection and dose adjustment of anti-epileptics is based on creatinine clearance, sedation potential, and co-morbidities of the patient [9,13,14]. Anti-epileptics are usually continued for two weeks to three months and then safely tapered off once symptoms and neuro-imaging findings resolve, as seizure recurrence and epilepsy appear to be rare in these patients [10,11,13]. In the setting of pregnancy, preeclampsia and eclampsia recommendations for treatment slightly differ [8,9,11,14]. Delivery of the baby and placenta is usually sufficient in the case of preeclampsia and eclampsia [9-11]. For patients with severe features, including persistent headaches, postpartum hypertension, and visual changes, the prompt administration of IV magnesium sulfate has been shown to be superior to other anti-epileptics [10,13,14]. The choice of antihypertensives depends on the stage of pregnancy, fetal health, as well as placental function and should be performed in conjunction with an obstetrician [13].

PRES is reversible in 70% to 90% of patients with prompt diagnosis and treatment [1,2,14]. Residual neurologic deficits with epileptiform disorders and motor deficits are some cited complications [1-3]. Serious complications, including intraparenchymal hemorrhage with mass effect, cerebral herniation from edema, and subarachnoid hemorrhage from endothelial dysfunction have been reported in less than 10% of cases [10,11,13]. Recurrent PRES has been reported in less than 5%, especially in patients that have nephrotic syndrome, HIV infection, and patients on chemotherapy [10,11,13]. Death from severe complications of cerebral infarction and extensive intra-cerebral hemorrhage is extremely rare but has been reported in cohort studies [1,10,13]. Most of the patients will require lifelong antihypertensives with avoidance of abrupt discontinuation of medications [9,10,14].

## Conclusions

Clinical signs, symptoms, and neuroimaging lesions lead to the diagnosis of posterior reversible encephalopathy syndrome. In our patient, the years of uncontrolled hypertension with long-standing cocaine use may have led to PRES. The exact pathophysiology of the syndrome is not clear; however, due to the inability of the posterior circulation of the brain to auto-regulate with acute changes in blood pressure resulting in hypo-perfusion and disruption of the blood-brain barrier with resultant vasogenic edema without infarction is the main theory. In this case, cocaine use has sympathomimetic mechanisms that can cause vasoconstriction and increase cardiac output, which can lead to hypertension. With each use of cocaine, chronic vasospasm results in vasoconstriction that can damage the blood vessel endothelium, which promotes atherosclerosis. Diagnosis is based on imaging, either CT or MRI. The treatment is to treat the underlying cause; for example, in this case, it was controlling the blood pressure with anti-hypertensives and blood pressure monitoring. To date, there are no clear data on the prevalence of cocaine-induced PRES, and we hope this case report will be used for further research. The prognosis of PRES is dependent on the underlying pathology, which is usually favorable. In this patient, after the blood pressure was controlled, symptoms improved, which was confirmed by MRI images. Hence, one can say that this was an important parameter in determining this patient's long-term prognosis.
